# Saccharification Potential of Transgenic Greenhouse- and Field-Grown Aspen Engineered for Reduced Xylan Acetylation

**DOI:** 10.3389/fpls.2021.704960

**Published:** 2021-09-07

**Authors:** Sivan Pramod, Madhavi Latha Gandla, Marta Derba-Maceluch, Leif J. Jönsson, Ewa J. Mellerowicz, Sandra Winestrand

**Affiliations:** ^1^Department of Forest Genetics and Plant Physiology, Umeå Plant Science Centre, Swedish University of Agricultural Sciences, Umeå, Sweden; ^2^Department of Chemistry, Umeå University, Umeå, Sweden

**Keywords:** *Populus tremula × tremuloides*, T89, genetic modification trees, field trial, saccharification, lignocellulose, cell wall acetylation, wood

## Abstract

High acetylation of xylan in hardwoods decreases their value as biorefinery feedstocks. To counter this problem, we have constitutively suppressed *RWA* genes encoding acetyl-CoA transporters using the *35S* promoter, or constitutively and wood-specifically (using the *WP* promoter) expressed fungal acetyl xylan esterases of families CE1 (*AnAXE1*) and CE5 (*HjAXE*), to reduce acetylation in hybrid aspen. All these transformations improved the saccharification of wood from greenhouse-grown trees. Here, we describe the chemical properties and saccharification potential of the resulting lines grown in a five-year field trial, and one type of them (*WP:AnAXE1*) in greenhouse conditions. Chemically, the lignocellulose of the field- and greenhouse-field-grown plants slightly differed, but the reductions in acetylation and saccharification improvement of engineered trees were largely maintained in the field. The main novel phenotypic observation in the field was higher lignification in lines with the *WP* promoter than those with the *35S* promoter. Following growth in the field, saccharification glucose yields were higher from most transformed lines than from wild-type (WT) plants with no pretreatment, but there was no improvement in saccharification with acid pretreatment. Thus, acid pretreatment removes most recalcitrance caused by acetylation. We found a complex relationship between acetylation and glucose yields in saccharification without pretreatment, suggesting that other variables, for example, the acetylation pattern, affect recalcitrance. Bigger gains in glucose yields were observed in lines with the *35S* promoter than in those with the *WP* promoter, possibly due to their lower lignin content. However, better lignocellulose saccharification of these lines was offset by a growth penalty and their glucose yield per tree was lower. In a comparison of the best lines with each construct, *WP:AnAXE1* provided the highest glucose yield per tree from saccharification, with and without pretreatment, *WP:HjAXE* yields were similar to those of WT plants, and yields of lines with other constructs were lower. These results show that lignocellulose properties of field-grown trees can be improved by reducing cell wall acetylation using various approaches, but some affect productivity in the field. Thus, better understanding of molecular and physiological consequences of deacetylation is needed to obtain quantitatively better results.

## Introduction

Plants are by far the most important contributors to biomass on Earth (accounting for ca. 450 of ca. 550 Gt C in total), mostly immobilized in cell walls of stems and tree trunks (Bar-On et al., 2018). Moreover, lignocellulose in tree trunks is considered one of the most promising renewable resources for production of sustainable materials, chemicals and energy with a balanced carbon cycle (Martinez-Abad et al., 2018). However, recalcitrance has strongly hindered conversion of this biomass into final desired products (McCann and Carpita, 2015; Yoo et al., 2017). There have been intense efforts to identify the main determinants of this recalcitrance and develop suitable varieties for biorefinery applications. Hardwoods (dicotyledonous tree species) include many fast-growing species (such as eucalypts, poplars, and aspens) that are suited for production of biorefinery feedstock in short-rotation plantations. However, relatively high acetyl contents in their wood reduce their attractiveness for biochemical conversion, a biorefining process that typically includes steps, such as hydrothermal pretreatment in acidic conditions, enzymatic saccharification, and microbial fermentation (Donev et al., 2018; Galbe and Wallberg, 2019). Acetylation affects all these steps. Hydrolysis of acetyl groups and formation of acetic acid during hydrothermal pretreatment contributes to acidification (Jönsson et al., 2013; Galbe and Wallberg, 2019), the remaining acetylation can affect susceptibility to enzymatic saccharification (Donev et al., 2018), and high acetic acid concentrations in the fermentation medium can inhibit microorganisms, such as yeast and bacteria, although low concentrations can result in higher product yields (Jönsson et al., 2013).

This has prompted intense research on the molecular pathways in dicotyledons responsible for acetylation of their lignocellulose, most of which is in the glucuronoxylan. The *O*-acetyl groups are present as side chains at positions 2 and 3 of the xylosyl backbone units, typically decorating alternate units at one or both of these positions (Busse-Wicher et al., 2014; Chong et al., 2014). This fraction of xylan has been called “the major xylan domain” and is thought to assume a two-screw confirmation allowing interaction with cellulose microfibrils by H-bonds (Grantham et al., 2017). A smaller fraction of xylan is substituted on consecutive xylosyl backbone units, which hinders interaction between the xylan and cellulose. This fraction could therefore occupy the space between cellulose microfibrils. The acetyl transferases involved in acetylation of xylan backbone have been identified as trichome birefringency-like (TBL) proteins (Xiong et al., 2013; Urbanowicz et al., 2014). Several closely related TBL genes of *Arabidopsis* are involved in addition of acetyl groups at specific positions in xylan backbone (Zhong et al., 2017), contribute to either the major or minor xylan domains, and cooperate with specific glucuronyl transferases (Grantham et al., 2017). Another protein involved is *At*AXY9, but its precise function is still unclear. The encoded protein has been suggested to interact with TBLs and reduced wall acetylation (RWA) Golgi transporters (Pauly and Ramirez, 2018) responsible for transporting acetyl-CoA (substrate for acetylation) from the cytosol to Golgi lumen (Zhong et al., 2020). The *RWA* gene family in *Populus* consists of two clades: clade I including *PtRWA-A* and *PtRWA-B* genes, similar to *AtRWA1* and *AtRWA3*, and clade II including *PtRWA-C* and *PtRWA-D* genes, similar to *AtRWA2*. Specific suppression of these clades in hybrid aspen has indicated that they both participate in acetylation of wood xylan (Pawar et al., 2017b).

Mutants with impairments in the genes responsible for xylan acetylation, which have significantly reduced lignocellulose acetyl contents, are frequently dwarf and have collapsed xylem vessels (an irregular xylem phenotype; Lee et al., 2011; Manabe et al., 2013). However, the dwarfism and collapsed xylem of severely affected *tbl29* (*eskimo*) mutants are reversed in strigolactone biosynthesis mutant *max4* background, indicating that the reduced acetylation is indirectly linked with dwarfism and uncoupling the two might be possible (Ramirez et al., 2018). Moreover, Pawar et al. (2016, 2017a,b) found that the dwarfism and xylem irregularity were not observed in lines with reductions in acetyl contents were below 40%.

Reducing acetylation by approx. 10–30% by either downregulating *PtRWA* genes (Pawar et al., 2017b) or expressing fungal acetyl xylan esterase (*AXE*) genes and targeting the encoded protein to cell walls (Pawar et al., 2016, 2017a; Wang et al., 2020) has led to promising 20–30% improvements in glucose yields in saccharification without pretreatment (Pawar et al., 2017a,b; Wang et al., 2020) or with either alkali or hot water pretreatment (Pawar et al., 2016) with no impairment in growth of the plants in greenhouse conditions. However, when tested in field conditions, plant productivity and foliage damage by beetles varied according to the transgene and promoter used (Derba-Maceluch et al., 2020). Use of the constitutive *35S* promoter generally led to worse field performance than use of the wood-specific promoter (*WP*; Ratke et al., 2015). Both plant growth and foliage health were compromised when *AXE* genes were expressed from the *35S* promoter. However, the plants with *WP*-driven *AXE* genes exhibited good growth and no higher leaf damage than wild-type (WT) plants. Thus, overall effects of reducing acetylation specifically in the wood seem promising, but it is essential to check that positive changes in wood properties observed in the greenhouse are maintained in field-grown plants. Therefore, we have analyzed stem lignocellulose of mentioned lines grown in the field and determined its properties in saccharification with and without acid pretreatment. *WP:AnAXE1* lines were grown in both the greenhouse and field conditions to evaluate effects of growth conditions on lignocellulose properties directly. We found that the lignocellulose properties of greenhouse- and field-grown plants were generally positively correlated, showing that the improved wood properties of *WP:AnAXE1*-expressing plants were maintained in the field conditions. This is a promising result, indicating that the main issue to test that could compromise the value of genetically modified plants with reduced acetylation may be their field productivity.

## Materials and Methods

### Biological Material

Transgenic lines used in this study are described by Derba-Maceluch et al. (2020). They were generated by *Agrobacterium* transformation from wild-type hybrid aspen (*Populus tremula* L. × *tremuloides* Michx. clone T89). Greenhouse-grown trees were harvested after 13 weeks of growth in previously described conditions (Wang et al., 2020). Developing wood was scraped from internodes 20–30 and used for RNA and acetyl esterase activity analysis. Internodes 33–50 were debarked, frozen, and freeze dried for 36 h, and their pith was removed before grinding. The field-grown material has been previously described (Derba-Maceluch et al., 2020). Wood was harvested after five growth seasons, from August 2014 to September 2018. A 30 cm long stem segment (15–45 cm from the ground) was removed from each of sets of 50% of the tallest trees of each transgenic line and WT trees, then dried at 60°C to constant weight before grinding.

### Transcript Level Analysis

Total RNA was extracted from developing wood and cDNA synthesized following Wang et al. (2020). Ubiquitin (Potri.005G198700) and tubulin (Potri.001G464400) were found to be the most stable of four reference genes tested by the RefFinder software (Xie et al., 2012) and used for normalization. Bio-Rad C1000 Touch CFX384 Real Time PCR Detection System was used. Expression levels of target genes were analyzed following Pfaffl (2001), and levels relative to those in the line most weakly expressing them are presented here. The primers used to amplify reference and targeted genes are shown in Supplementary Table S1.

### Acetyl Esterase Activity

Developing wood isolated as above was ground in liquid nitrogen in a mortar. Soluble proteins were extracted for 1 h at 4°C with stirring in 50 mM sodium phosphate buffer (pH 5.0) containing 0.2 mM EDTA, 4% polyvinylpyrrolidone (PVP) m.w. 360,000, and 1 mM dithiothreitol, and then, the soluble fraction was collected by centrifugation (20,000 *g*, 10 min). The resulting pellet was resuspended in the same buffer supplemented with 1 M NaCl, and incubated for 1 h at 4°C with stirring. Wall-bound proteins were collected after centrifugation as above. All buffers contained cOmplete^™^ Protease Inhibitor Cocktail (Roche). Protein concentration was determined using Bradford assay (Bradford, 1976).

Acetyl esterase activity in extracts was determined as described by Chung et al. (2002), using 300 μl assay mixtures containing sodium acetate buffer (10 mM, pH 5.0), 1 μmole of 4-nitrophenyl acetate substrate, and 10 μg of extracted soluble or wall-bound proteins. After incubation at 37°C for 3 h, the 4-nitrophenol (4NP) released by esterase activity was determined by measuring absorbance at 405 nm, and using a standard 4NP curve for calibration. Specific activity was expressed as μmols of 4NP released per mg protein per h at 37°C.

### Cell Wall Compositional Analysis

#### Preparation of Wood Powder

Wood from trees grown in both field and greenhouse conditions was ground to a rough powder using an SM 300 Cutting Mill with a 2 mm sieve (Retsch, Haan, Germany). The rough powder was then sieved using an AS 200 vibratory sieve shaker (Retsch) and divided into the following particle size fractions: < 50, 50–100, 100–500, and > 500 μm. Wood powder of each size fraction from sets of three greenhouse-grown trees and four field-grown trees was then pooled. Three such pools of each size fraction were prepared from the greenhouse-grown material as biological replicates, as well as three and six, respectively, of the transgenic and WT field-grown material.

#### Py-GC/MS

Portions (50 ± 10 μg) of wood powder of the < 50 μm particle size fraction were subjected to pyrolysis gas chromatography combined with mass spectrometry (Py-GC/MS), as previously described (Gandla et al., 2015). For this, we used a PY-2020iD pyrolyzer equipped with an AS-1020E autosampler (Frontier Lab, Japan) connected to a 7890A/5975C GC/MS system (Agilent Technologies AB, Sweden). The pyrolysate was separated and analyzed according to Gerber et al. (2012).

#### Preparation of AIR and Acetyl Content Analysis

Alcohol-insoluble residue (AIR) was obtained from wood powder of the < 50 μm particle size following Gandla et al. (2015). Portions (300 μg) of AIR were saponified by incubation in 0.5 M NaOH at room temperature for 1 h. The resulting solution was neutralized with 1 M HCl and its acetic acid content was determined using a K-ACET Megazyme kit (Megazyme, Wicklow, Ireland).

#### Matrix Monosaccharide and Cellulose Analysis in Starch-Free Residue

Portions (5 mg) of AIR were treated with α-amylase and α-amyloglucosidase (both from Roche, United States) and dried, as described by Gandla et al. (2015). Portions (500 μg ± 10%) of dry, destarched AIR and 30 μg of inositol (internal standard), together with standards of nine monosaccharides (Ara, Rha, Fuc, Xyl, Man, Gal, Glc, GalA, and GlcA, each at 10, 20, 50, and 100 μg), were prepared. They were methanolyzed and derivatized; then, the silylated monosaccharides were separated using the GC/MS system mentioned above, following Gandla et al. (2015). Raw data files from the GC/MS analysis were converted to CDF format by Agilent Chemstation Data Analysis (Version E.02.00.493) and exported to R software (Version 3.0.2; R Development Core Team). R software was also used for data pretreatment procedures, such as baseline correction and chromatogram alignment, time-window setting, and multivariate curve resolution processing, followed by peak identification. 4-*O*-methylglucuronic acid was identified according to Chong et al. (2013). Contents of monosaccharide units per unit weight (hereafter per g, for convenience) of destarched AIR were calculated, assuming that they were in a polymeric form.

Portions (3 mg) of destarched AIR were also used for crystalline cellulose analysis following Gandla et al. (2015).

#### Analysis of Soluble Sugars and Starch

Soluble sugars were extracted with 80 and 70% ethanol as previously described (Stitt et al., 1989) from 30 mg portions of wood powder (< 50 μm particles); then, the Glc, Frc, and Suc contents were determined by coupled enzyme-based spectrophotometric assay using NADP^+^ reduction (Roach et al., 2012).

Starch was analyzed in 20 mg portions of the residues following ethanol extraction, by gelatinization and enzymatic degradation (Hendriks et al., 2003; Smith and Zeeman, 2006). For gelatinization, samples were suspended in 0.1 M NaOH (40 μl/mg of residue) and incubated at 95°C for 30 min. After cooling, 8 μl of 0.1 M sodium acetate/NaOH buffer was added per mg of residue. The mixtures were thoroughly mixed; then, 40 μl aliquots were transferred to a 2 mL tube and treated with 110 μl of enzyme mix containing 0.5 U alpha-amylase and 6 U of alpha-amyloglucosidase in 50 mM sodium acetate buffer (pH 4.9). Samples were incubated at 37°C overnight. Negative controls were processed in the same way except that buffer was used *in lieu* of sample. Portions (50 μl) of the samples or control mixtures were then incubated with 160 μl of buffer containing HEPES [4-(2-hydroxyethyl)-1-piperazineethanesulfonic acid; 100 mM, pH 7.0], MgCl_2_ (3 mM), NADP (1 mM), ATP (2.5 mM; all from Sigma, Aldrich, United States), and glucose-6-P-dehydrogenase (0.625 U; Roche, Germany) at 37°C. Hexokinase (1 U, supplied by Roche, Germany) was added to each mixture and the starch degradation kinetics were monitored during the period 20–40 min after the addition using an Epoch™ microplate spectrophotometer (BioTek, Germany). Finally, glucose concentrations of the samples were estimated from interpolation of the recorded absorbance at 340 nm to a glucose standard curve.

### Analytical Saccharification

Portions of dry wood powder (50 mg, 0.1–0.5 mm particles) were subjected to analytical saccharification after moisture analysis using an HG63 moisture analyzer (Mettler-Toledo), following Gandla et al. (2021). Pooled samples of the lines (prepared as described above) were analyzed in triplicate. Briefly, without or after acid pretreatment (using an Initiator single-mode microwave instrument supplied by Biotage, Uppsala, Sweden), they were subjected to enzymatic hydrolysis at 45°C using 5 mg of Cellic CTec-2 liquid enzyme preparation (Sigma-Aldrich, St. Louis, MO, United States). Sub-samples were collected after incubation for two and 72 h. The glucose production rate from sub-samples collected after 2 h was estimated using an Accu-Chek^®^ Aviva glucometer (Roche Diagnostics Scandinavia AB, Bromma, Sweden) after calibration with a set of glucose standard solutions. For samples collected after 72 h, yields of monosaccharides (including arabinose, galactose, glucose, xylose, and mannose) were quantified using an Ion Chromatography System ICS-5000 high-performance anion-exchange chromatography system with pulsed amperometric detection (Dionex, Sunnyvale, CA, United States; Wang et al., 2018).

Glucose yields per tree were estimated from the trees’ stem volume, obtained from their stem diameter and height (Derba-Maceluch et al., 2020), and average estimated basic specific gravity (s.g.) of the wood (dry weight per unit green volume). The s.g. values applied were 0.58 ± 0.034 (SE) g/cm^3^ for field-grown WT (T89) trees, derived from analyses of 32 trees, and 0.27 ± 0.003 (SE) for both WT and *35S:AnAXE1* trees grown in the greenhouse, derived from analyses of seven and eight trees, respectively.

### Porosity Analysis

The surface area of sieved wood powder with 100–500 μm particles was analyzed with a single-point Tristar 3,000 BET Brunauer-Emmett-Teller Analyzer (Micromeritics, Atlanta, GA, United States). Samples were subjected to degassing using a SmartPrep Degasser (Micromeritics) prior to the analysis to remove nonspecific adsorbents. The instrument provides estimates of sample’s surface areas from the multilayered physical adsorption of nitrogen gas molecules.

### Statistical Analysis

Univariate analyses were performed using JMP^®^, Version Pro 14 (SAS Institute Inc., Cary, NC, 1989–2021). Multivariate analyses were performed using Orthogonal Projections to Latent Structures (OPLS) and Variable Influence on Projection (VIP; Galindo-Prieto et al., 2014) determinations obtained with SIMCA 16.0.17928 (Sartorius Stedim Data Analytics AB). The data used in the analyses were subjected to UV scaling, and variables were considered important for predicting glucose yields in saccharification when VIP_pred_ > 1 and VIP_tot_−CI_95_ > 0, where VIP_pred_ is predictive VIP, VIP_tot_ is total VIP, and CI_95_ is the 95% confidence interval.

## Results

### Characterization of Transgenic Lines Expressing *WP:AnAXE1* Grown in the Greenhouse

**Figure 1:**
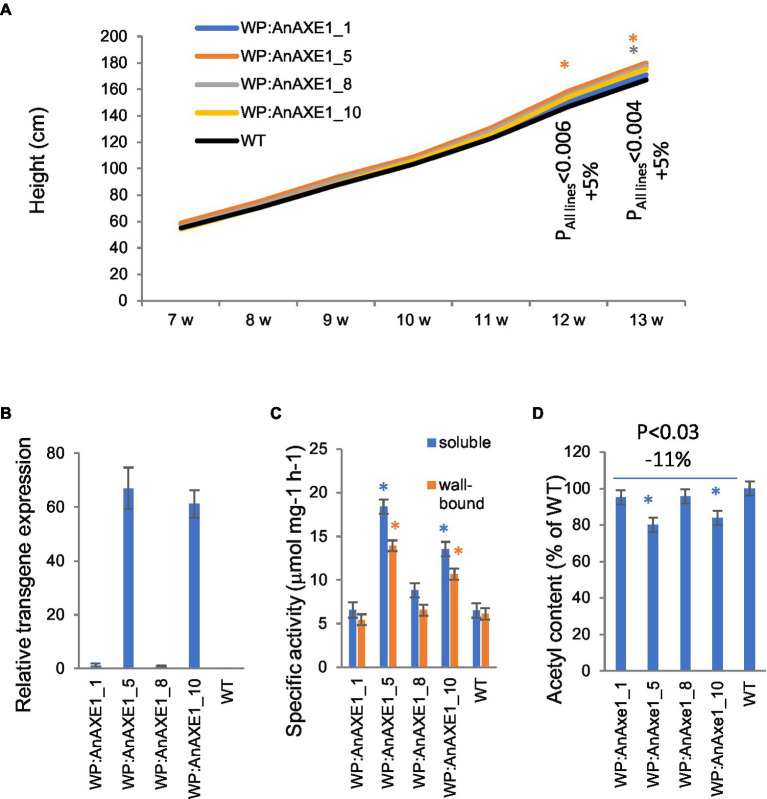
Phenotype of transgenic aspen grown in the greenhouse expressing wood-promoter driven *AnAXE1*. **(A)** Height growth. Means for 10 trees per line. *SE* varied between 1.4 and 3.1% of the means (not shown for clarity). The values of *p* indicate the significance of differences between all the transgenic lines and wild-type (WT) plants (post-ANOVA contrast analysis). **(B)** Transgene expression in developing wood. Means ± *SE* and *N* = 4 biological replicates. Values are relative to those of the most weakly expressing line (8). No transcripts were detected in WT plants. **(C)** Specific acetyl esterase activity in soluble and wall-bound protein fractions extracted from developing wood of transgenic and WT plants. Means ± *SE* from three independent experiments. **(D)** Acetyl content of the wood of transgenic and WT plants. Means ± *SE* of three biological replicates. Significant differences according to ANOVA followed by Dunnett’s test (*p* < 5%) are indicated by asterisks.

Transgenic lines carrying *WP:AnAXE1* included in the field experiment (Derba-Maceluch et al., 2020) have not been previously characterized in detail. These lines were therefore grown in the greenhouse and characterized in terms of transgene expression level, esterase activity, acetyl content, and wood cell wall properties to enable later comparisons between field- and greenhouse-grown material. Four transgenic lines selected from approx. 20 generated based on *in vitro* expression level were included in the study. The transgenic plants were slightly taller than WT by the end of the 13-week experiment (Figure 1A). Levels of transcripts of transgenes in developing wood were much higher in lines 5 and 10 than in lines 1 and 8 (Figure 1B). The acetyl esterase activity of soluble and wall-bound proteins extracted from developing wood was correspondingly higher in lines 5 and 10 (Figure 1C). The acetyl content of the wood in transgenic lines was significantly lower than in WT plants in lines 5 and 10 by 20 and 16%, respectively (Figure 1D), within the range observed in other transgenic lines tested in current field experiment (Ratke et al., 2015; Pawar et al., 2017a,b; Wang et al., 2020). Overall, plants of all tested transgenic lines had ca. 11% lower acetyl contents than WT counterparts (*p* < 0.03).

Chemical analysis of wood from *WP:AnAXE1* plants by Py-GC/MS did not reveal significant differences in carbohydrate or lignin contents compared to WT plants except for a small reduction in S/G ratio (Supplementary Table S2).

### Sugar Yields and Glc Production Rates of Wood of *WP:AnAXE1* Lines Grown in the Greenhouse

**Figure 2:**
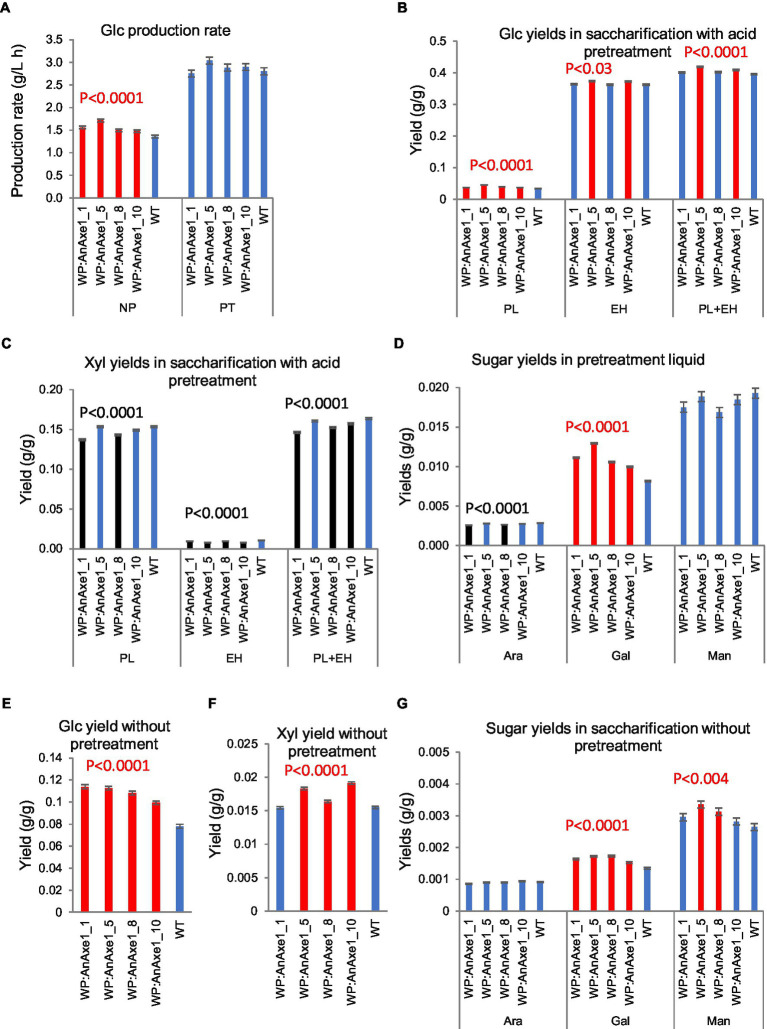
Saccharification parameters of developing wood from transgenic greenhouse-grown aspen expressing wood-promoter driven *AnAXE1* (*WP:AnAXE1*). **(A)** Glucose production rates in saccharification without (NP) and with (PT) acid pretreatment. Yields of glucose **(B)**, xylose **(C)**, and minor sugars **(D)** in saccharification with acid pretreatment. PL, pretreatment liquid; EH, enzymatic hydrolysis; and PL + EH, total sugar yield. Yields of glucose **(E)**, xylose **(F)**, and minor sugars **(G)** in saccharification without pretreatment. Means ± *SE* of three biological replicates. Significant differences according to ANOVA followed by Dunnett’s test (*p* < 5%) are indicated by color (red – higher and black – lower, than WT parameters). The values of *p* show the significance of differences between all the lines carrying indicated constructs and WT plants (post-ANOVA contrast, shown when < 5%).

Saccharification analysis of greenhouse-grown *WP:AnAXE1* lines revealed differences in sugar yields per g wood and glucose production rates (GPR) compared to WT plants, some of which were observed in all transgenic lines, even those with weak transgene expression (Figure 2; Supplementary Table S2). All transgenic lines provided significantly higher than WT GPR and yields, and galactose yields, in saccharification with no pretreatment (NP; Figures 2A,E,G), glucose and galactose yields in pretreatment liquid (PL; Figures 2B,D), and galactose yields in NP (Figure 2G). Total and enzymatic hydrolysis glucose yields from wood of most highly expressing lines were improved after acid pretreatment, and wood from other lines tended to provide higher total glucose yields in saccharification with pretreatment (PT; Figure 2B).

Mannose yields in NP also tended to be higher from the transgenic plants (Figure 2G). Xylose yields were differentially affected in PT and NP. In PT, the transgenics tended to yield less xylose in both the PL and enzymatic hydrolysates (EH; Figure 2C), whereas the yields were higher in NP from transgenic lines than from WT plants (Figure 2F).

These analyses revealed that effects of *WP:AnAXE1* on yields depended on the sugar; in most cases, effects were positive for six-carbon sugars and negative for five-carbon sugars. Positive effects on glucose yields were more substantial in NP, as reported for other transgenic aspen plants with reduced acetyl contents (Pawar et al., 2017a,b; Wang et al., 2020).

### Correlations of Woody Biomass Features With Saccharification Yields of *WP:AnAXE1* Lines Grown in the Greenhouse

To elucidate causes of the improved six-carbon sugar yields and variable xylose yields in saccharification, we analyzed in more detail the biomass of the two most highly transgene-expressing *WP:AnAXE1* lines: 5 and 10. The crystalline cellulose content was not altered in these lines, but their matrix sugar composition differed in some respects from WT plants. Their xylose and 4-*O*-Me-GlcA contents were 4 and 15% lower, respectively, indicating reductions in glucuronoxylan contents, and galactose contents almost 50% higher, with no corresponding increase in arabinose units, indicative of increases in galactan content (Table 1; Supplementary Table S3). Interestingly, their matrix glucose unit contents were increased without a corresponding increase in mannose units, which can be attributed to amorphous cellulose or callose. Contributions of starch or soluble glucose can be excluded since the analyzed material was treated with alcohol and amylase. Moreover, no significant differences were detected in soluble sugars and starch contents of untreated wood powder from transgenic and WT plants (Supplementary Table S3). Reductions in matrix xylose content and xylan chain lengths have been previously observed in several other plants with reduced acetyl contents and attributed to higher accessibility of deacetylated xylan to native xylanases and trans-xylanases of the GH10 family (Pawar et al., 2017a,b; Wang et al., 2020). In contrast, high increases in matrix galactose and glucose contents are indicative of tension wood in transgenic plants (Mellerowicz and Gorshkova, 2012; Gorshkova et al., 2015). Thus, the changes in xylose and galactose yields in PT can be explained by compositional changes in the matrix polysaccharides. However, since no change in matrix mannose units contents was detected in transgenic plants (Table 1), the higher mannan yields in NP are probably related to a change in cell wall architecture. To support the hypothesis that cell wall architecture was altered in the transgenic plants, we analyzed their lignocellulose porosity by the BET technique as described by Wang et al. (2020), and found it was ca. 30% higher in both transgenic lines than in WT plants (Table 1), similar to the previously observed effect of decreasing xylan acetylation in transgenic aspen expressing *WP:HjAXE* (Wang et al., 2020).

**Table 1:** Wood traits of selected *WP:AnAXE1* lines grown in the greenhouse.

Traits	*WP:AnAXE1_5*	*WP:AnAXE1_10*	WT
Ara (Mol %)	0.49 ± 0.06	0.45 ± 0.04	0.37 ± 0.02
Rha (Mol %)	1.24 ± 0.09	1.26 ± 0.04	1.22 ± 0.004
Fuc (Mol %)	0.06 ± 0.01	0.05 ± 0.001	0.04 ± 0.005
Xyl (Mol %)	**77.7** ± 0.62^*^	78.7 ± 0–89	**81.1** ± 0.6^*^
Man (Mol %)	3.93 ± 0.11	3.69 ± 0.11	4.12 ± 0.15
MeGlcA (Mol %)	0.69 ± 0.02	0.69 ± 0.02	**0.81** ± 0.05^*^
Gal (Mol %)	**0.99** ± 0.07^*^	0.89 ± 0.09	**0.63** ± 0.05^*^
GalA (Mol %)	1.61 ± 0.06	1.88 ± 0.18	1.76 ± 0.04
Glc (Mol %)	**13.19** ± 0.83^*^	12.29 ± 0.57	**9.83** ± 0.67^*^
GlcA (Mol %)	0.05 ± 0.002	0.06 ± 0.01	0.05 ± 0.01
Total sugar yield (mg/g)	188 ± 4.05	184 ± 3.24	184 ± 2.14
Cellulose (mg/g)	484 ± 21	513 ± 22	522 ± 9
BET surface area m^2^/g	2.19 ± 0.15	**2.46** ± 0.17^*^	**1.79** ± 0.06^*^

Means ± *SE*, *N* = 3. Emboldened values for transgenic lines significantly differ from WT values (Dunnett’s test, ^*^p < 5%) and emboldened WT values significantly differ from values for both transgenic lines taken together (post-ANOVA contrast, ^*^p < 5%).

### Chemical Composition of Lignocellulose in Transgenic Lines Grown in the Field

**Figure 3:**
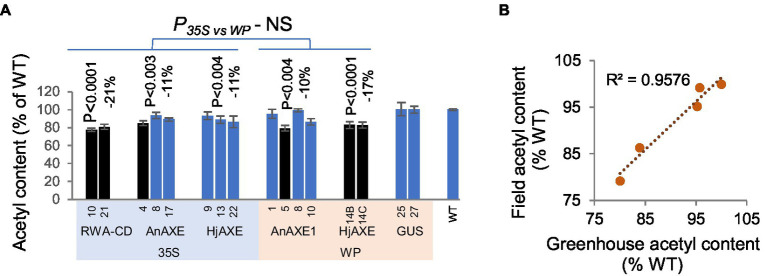
Acetyl content of lignocellulose biomass from stems of transgenic five-year-old field-grown aspen. **(A)** Acetyl content, in percentages of wild-type (WT) levels. Means ± *SE*. Significant downregulation, according to ANOVA followed by Dunnett’s test (*p* < 5%), is indicated by black color. The values of *p* show the significance of differences between all the lines carrying indicated constructs and WT plants (post-ANOVA contrast, shown when < 5%). There was no significant differences (NS) in effects of the promoters in acetyl content according to ANOVA *F*-tests. **(B)** Correlation of acetyl contents between field-grown (“Field”) and greenhouse-grown (“Greenhouse”) *WP:AnAXE1* lines.

To determine if the reduced acetylation of transgenic lines was maintained in the field conditions, we analyzed the acetyl content in the stems of 5-year-old field-grown trees and found that it was reduced in all lines according to the contrast analysis (Figure 3A). The strongest reduction (21%) was observed in lines with suppressed aspen *RWA-C* and *RWA-D* (*RWA-CD*) genes homologous to *AtRWA2* (Pawar et al., 2017b). There was no significant difference between lines with the *35S* and *WP* promoters. Among lines grown in both the field and greenhouse, a strong correlation was observed between their acetyl contents under the two conditions (Figure 3B).

To obtain an overview of the chemical composition of lignocellulose of transgenic field-grown trees, ground stem material was analyzed by Py-GC/MS (Table 2; Supplementary Tables S4 and S5). This revealed few differences between the genetically modified and WT plants. *35S:AnAXE1* lines had lower S/G ratios, *WP:HjAXE* lines had higher lignin and lower carbohydrate contents, and *35S:HjAXE* lines had lower H lignin contents than WT counterparts. In addition, in a comparison of *HjAXE* and *AnAXE1* lines, we found that *HjAXE*-expressing lines with either promoter had higher lignin S/G ratios and contents, due to accumulation of both G and S subunits, whereas *AnAXE1*-expressing lines had higher H lignin contents (Table 2). Thus, *AXE* expression seemed to induce some changes in lignin content and composition in the field-grown trees.

**Table 2:** Chemical composition, S/G and C/L ratios, and lignin contents, according to pyrolysis-gas chromatography mass spectrometry, of lignocellulose of lines carrying indicated constructs and WT plants grown in the field.

Traits	*35S:RWA-CD*	*35S:AnAXE1*	*35S:HjAXE*	*WP:AnAXE1*	*WP:HjAXE*	*WP:GUS*	WT	*35S/WP*	*F_35S vs. WP_*	*AnAXE1/HjAXE*	*F_AnAXE1 vs. HjAXE_*
C (%)	69.6 ± 0.3	69.0 ± 0.2	**69.6 ± 0.2** ^*^	69.0 ± 0.2	**67.6 ± 0.3** ^*^	68.7 ± 0.3	68.7 ± 0.3	1.01	^***^	1.01	^**^
G (%)	8.98 ± 0.14	9.44 ± 0.11	9.48 ± 0.11	9.30 ± 0.10	**9.91 ± 0.14** ^**^	9.65 ± 0.14	9.19 ± 0.14	0.98		0.96	^***^
S (%)	8.16 ± 0.19	8.30 ± 0.15	9.29 ± 0.15	8.96 ± 0.13	9.30 ± 0.19	9.30 ± 0.19	8.77 ± 0.19	0.95	^**^	0.92	^***^
H (%)	2.81 ± 0.11	2.80 ± 0.09	**2.32 ± 0.09** ^**^	2.69 ± 0.08	2.82 ± 0.11	2.56 ± 0.11	2.80 ± 0.11	0.96		1.07	^*^
P (%)	3.24 ± 0.07	3.24 ± 0.06	3.01 ± 0.06	3.00 ± 0.05	3.13 ± 0.07	2.96 ± 0.07	3.16 ± 0.07	1.04	^*^	1.02	
S/G (ratio)	0.910 ± 0.012	**0.880 ± 0.010** ^***^	0.979 ± 0.010	0.964 ± 0.009	0.938 ± 0.012	0.963 ± 0.012	0.955 ± 0.012	0.97	^**^	0.96	^***^
C/L (ratio)	3.01 ± 0.04	2.91 ± 0.03	2.89 ± 0.03	2.89 ± 0.03	**2.69 ± 0.01** ^**^	2.81 ± 0.04	2.87 ± 0.04	1.04	^**^	1.04	^***^
L (%)	23.19 ± 0.23	23.78 ± 0.19	24.11 ± 0.19	23.96 ± 0.23	**25.16 ± 0.23** ^**^	24.47 ± 0.23	23.92 ± 0.23	0.97	^***^	0.97	^***^

* *p* < 5%;

** *p* < 1%;

*** *p* < 0.1%.

Means ± *SE*, *N* = 3 for transgenic lines and six for WT. Emboldened values significantly differ from WT values according to Dunnett’s test. Comparison of lines with 35S (35S:RWA-CD, 35S:AnAXE1, and 35S:HjAXE) and WP (WP:AnAXE1 and WP:HjAXE) promoters and AnAXE1 (35S:AnAXE1 and WP:AnAXE1) and HjAXE (35S:HjAXE and WP:HjAXE) transgenes. The significance of differences was assessed by ANOVA F-tests. C, carbohydrates; S, syringyl lignin units; G, guaiacyl lignin units; H, p-hydroxyphenyl lignin units; P, phenolics; and L, lignin (S + G + H + P).

Since growth of the transgenic lines is affected by the promoter used for the deacetylation (Derba-Maceluch et al., 2020), we tested effects of constructs with the two promoters on wood chemistry and detected small but highly significant differences (Table 2). Lines in which the transgene was ectopically expressed had more carbohydrates (C) and phenolic compounds (P) and less total and S lignin than lines expressing it solely in wood.

Although there was no significant difference between the *WP:AnAXE1* and WT lines in lignocellulose chemical composition, according to Py-GC/MS analysis, there were significant differences between the lignocellulose contents of material (of both lines) grown in the greenhouse and field (Table 3). The most striking difference was in phenolic compounds, which were an order of magnitude more abundant in the field-grown material, possibly due to the presence of bark in the lignocellulose samples from the field.

**Table 3:** Chemical composition, S/G and C/L ratios, and lignin contents, according to pyrolysis-gas chromatography mass spectrometry, of lignocellulose of *WP:AnAXE1* and WT lines grown in the field and greenhouse.

		*WP:AnAXE1*				WT	*P_field vs. greenhouse_* > *F*
		Line 1	Line 5	Line 8	Line 10		
C (%)	*field*	68.6 ± 0.5	69.3 ± 0.5	69.0 ± 0.5	69.3 ± 0.5	68.7 ± 0.4	< 0.0001
	*greenhouse*	71.7 ± 0.5	72.2 ± 0.5	72.0 ± 0.5	72.1 ± 0.5	72.1 ± 0.5	
G (%)	*field*	9.6 ± 0.2	8.8 ± 0.2	9.2 ± 0.2	9.5 ± 0.2	9.2 ± 0.2	< 0.0001
	*greenhouse*	10.1 ± 0.2	10.1 ± 0.2	9.9 ± 0.2	9.9 ± 0.2	9.8 ± 0.2	
S (%)	*field*	9.0 ± 0.4	9.1 ± 0.4	8.5 ± 0.4	9.2 ± 0.4	8.8 ± 0.3	< 0.0001
	*greenhouse*	11.5 ± 0.4	11.3 ± 0.4	11.4 ± 0.4	11.1 ± 0.4	11.4 ± 0.4	
H (%)	*field*	2.7 ± 0.1	2.7 ± 0.1	3.0 ± 0.1	2.4 ± 0.1	2.8 ± 0.1	0.23
	*greenhouse*	2.6 ± 0.1	2.4 ± 0.1	2.6 ± 0.1	2.7 ± 0.1	2.7 ± 0.1	
P (%)	*field*	3.1 ± 0.1	3.0 ± 0.1	3.0 ± 0.1	2.9 ± 0.1	3.2 ± 0.0	< 0.0001
	*greenhouse*	0.2 ± 0.1	0.2 ± 0.1	0.2 ± 0.1	0.2 ± 0.1	0.2 ± 0.1	
S/G (ratio)	*field*	0.9 ± 0.0	1.0 ± 0.0	0.9 ± 0.0	1.0 ± 0.0	1.0 ± 0.0	< 0.0001
	*greenhouse*	1.1 ± 0.0	1.1 ± 0.0	1.2 ± 0.0	1.1 ± 0.0	1.2 ± 0.0	
C/L (ratio)	*field*	2.8 ± 0.1	2.9 ± 0.1	2.9 ± 0.1	2.9 ± 0.1	2.9 ± 0.1	0.06
	*greenhouse*	3.0 ± 0.1	3.0 ± 0.1	3.0 ± 0.1	3.0 ± 0.1	3.0 ± 0.1	
L (%)	*field*	24.4 ± 0.6	23.7 ± 0.6	23.8 ± 0.6	24.0 ± 0.6	23.9 ± 0.4	0.62
	*greenhouse*	24.5 ± 0.6	24.0 ± 0.6	24.2 ± 0.6	24.0 ± 0.6	24.0 ± 0.6	

Note that the field-collected lignocellulose, but not the greenhouse-collected lignocellulose, included bark. Means ± *SE*, *N* = 3 for transgenic lines and six for WT. The significance of the conditions (field vs. greenhouse) for the considered traits according to ANOVA F-tests is shown. Line effects were not significant. C, carbohydrates; S, syringyl lignin units; G, guaiacyl lignin units; H, p-hydroxyphenyl lignin units; P, phenolics; and L, lignin (S + G + H + P).

### Sugar Yields and Glc Production Rates From Lignocellulose From Transgenic Trees Grown in the Field

**Figure 4:**
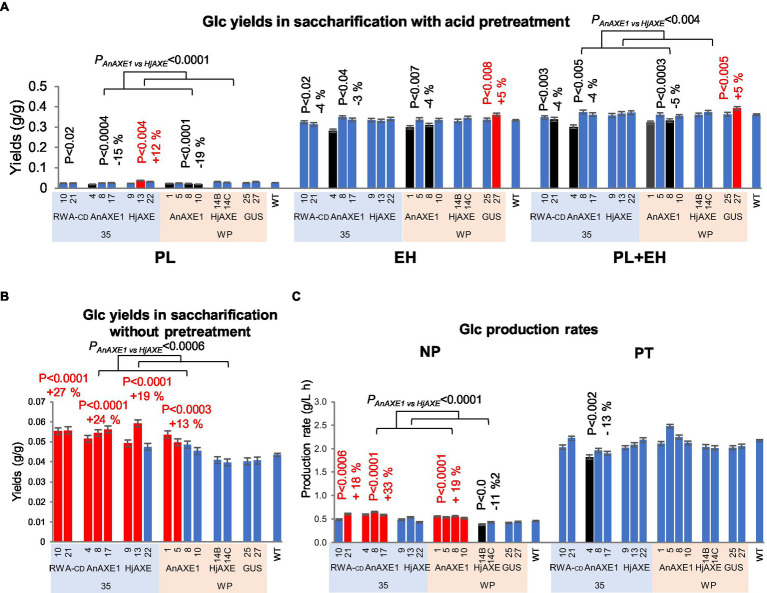
Saccharification parameters of transgenic five-year-old field-grown aspen (glucose yields and production rates). **(A)** Glucose yields in saccharification with acid pretreatment. PL, pretreatment liquid; EH, enzymatic hydrolysis; and PL + EH, total sugar yield. **(B)** Glucose yields in saccharification without pretreatment. **(C)** Glucose production rates. NP, not pretreated; PT, pretreated. Means ± *SE*. Significant differences according to ANOVA followed by Dunnett’s test (*p* < 5%) are indicated by color (red – higher and black – lower, than WT parameters). The values of *p* show the significance of differences between all the lines carrying indicated constructs and WT plants (post-ANOVA contrast, shown when < 5%). Effects of the transgene (*AnAXE1 vs. HjAXE*) were assessed by ANOVA *F*-tests.

Wood from transgenic lines with reduced acetylation did not provide better glucose yields in PT than wood from WT plants (Figure 4; Supplementary Tables S4 and S5). Material from all lines except *35S:HjAXE* and *WP:HjAXE* lines provided slightly (4–5%) lower glucose yields, in both PL and EH (Figure 4A). Glucose production rates (GPR) from *35S:AnAXE1* material were also lower (Figure 4C). In a comparison of *AnAXE1* and *HjAXE* material, the latter performed better in PT in terms of both glucose yields in PL and total yields. In contrast, in NP material from all tested transgenic lines except *WP:HjAXE* lines (Figure 4B) provided higher glucose yields than WT material (by 13–27%), and higher GPR was obtained with material from all the transgenics except *35SHjAXE* and *WP:HjAXE* (Figure 4C). This indicates that the acid pretreatment at the applied severity level largely eliminates recalcitrance caused by acetylation, but *AnAXE1* had more desirable effects than *HjAXE* on glucose yields and GPR in NP.

**Figure 5:**
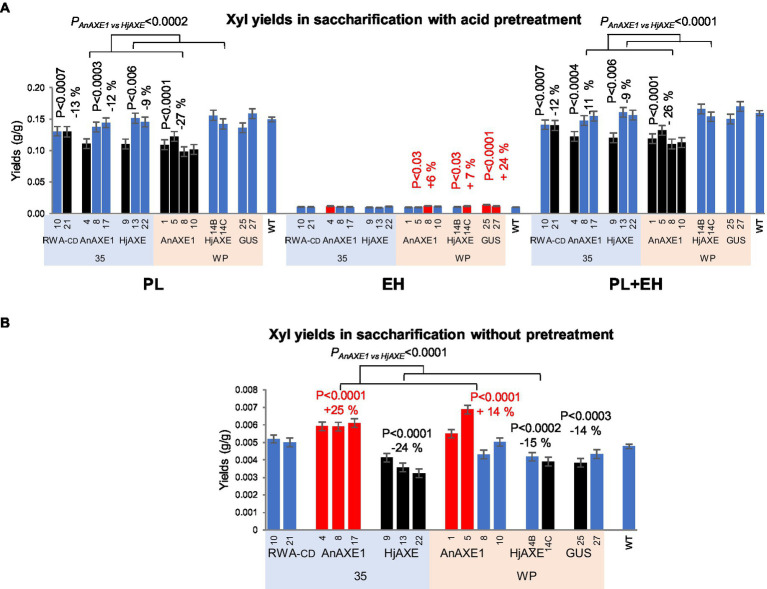
Saccharification parameters of transgenic five-year-old field-grown aspen (xylose yields). **(A)** Xylose yields in saccharification with acid pretreatment. PL, pretreatment liquid; EH, enzymatic hydrolysis; and PL + EH, total sugar yield. **(B)** Xylose yields in saccharification without pretreatment. Means ± *SE*. Significant differences according to ANOVA followed by Dunnett’s test (*p* < 5%) are indicated by color (red – higher and black – lower, than WT parameters). The values of *p* show the significance of differences between all the lines carrying indicated constructs and WT plants (post-ANOVA contrast, shown when < 5%). Effects of the transgene (*AnAXE1 vs. HjAXE*) were assessed by ANOVA *F*-tests.

Xylose yields obtained in PT largely reflected the yields obtained in PL and were lower from samples of all lines with reduced acetylation than from WT samples, except those carrying the *WP:HjAXE* construct (Figure 5A; Supplementary Tables S4 and S5). The lowest yields (26% lower than from WT samples) were obtained from samples of *WP:AnAXE1* lines. In addition, without pretreatment, the xylose yields were differentially affected by the two AXEs: increased by *AnAXE1* and reduced by *HjAXE* (Figure 5B; Supplementary Tables S4 and S5). The most pronounced effects were observed in lines with constitutive transgene expression (net changes of +25 and −24%, respectively). Xylose yields in NP were also somewhat affected in *WP:GUS* lines.

**Figure 6:**
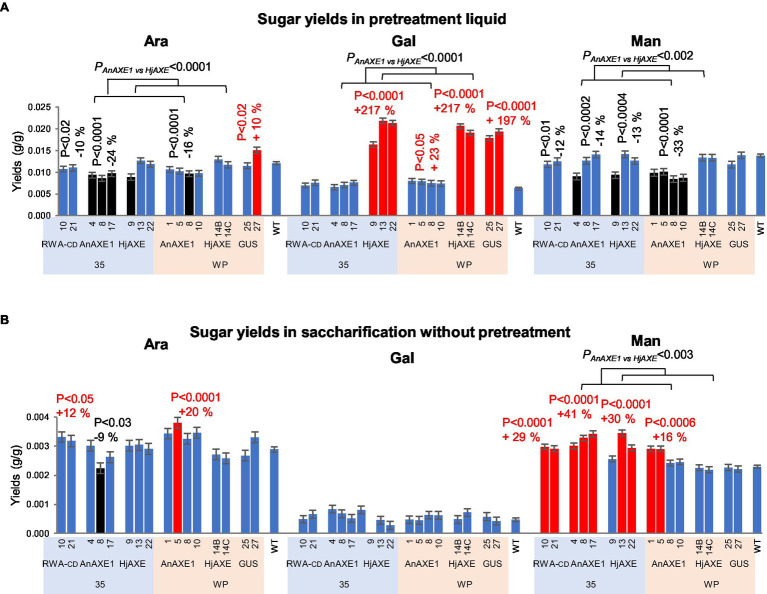
Saccharification parameters of transgenic five-year-old field-grown aspen (minor sugar yields). **(A)** Arabinose, galactose, and mannose yields in saccharification with acid pretreatment. The sugars were only detected in the pretreatment liquid. **(B)** Arabinose, galactose, and mannose yields in saccharification without pretreatment. Means ± *SE*. Significant differences according to ANOVA followed by Dunnett’s test (*p* < 5%) are indicated by color (red – higher and black – lower, than WT parameters). The values of *p* show the significance of differences between all the lines carrying indicated constructs and WT plants (post-ANOVA contrast, shown when < 5%). Effects of the transgene (*AnAXE1 vs. HjAXE*) were assessed by ANOVA *F*-tests.

Yields of minor sugars were significantly affected by expression of the constructs in the transgenic lines. In PT, the minor sugars were released during the pretreatment, and none were detected following subsequent enzymatic hydrolysis. Arabinose and mannose yields were reduced by expression of all acetyl-reducing constructs except *WP:HjAXE*, while galactose yields were increased up to 2-fold by *HjAXE* expression (in all lines), and 23% by the *WP:AnAXE1* construct (Figure 6A; Supplementary Tables S4 and S5). In addition, transformation with the *WP:GUS* construct resulted in a 2-fold increase in galactose yield. Without pretreatment, yields of mannose were elevated from material of most lines with reduced acetylation contents (except *WP:HjAXE* lines), and yields of arabinose from lines with *35S:RWA-CD* and *35S:AnAXE1* constructs (Figure 6B).

### Comparison of Saccharification of Field- and Greenhouse-Grown Material (Correlation Analyses)

**Figure 7:**
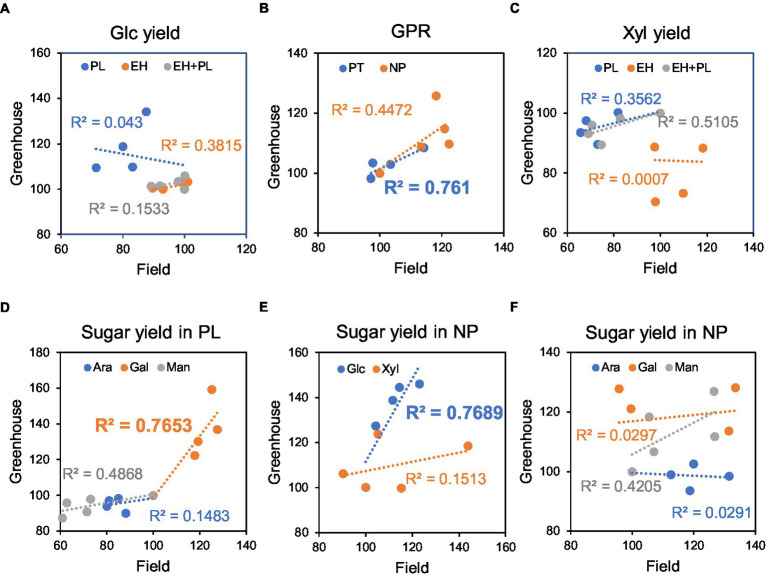
Relationships between saccharification yields and GPR from material of plants grown in the field and greenhouse. Data for developing wood of four transgenic (*WP:AnAXE1*) and wild-type (WT) lines in percentages of WT values. Coefficients of linear correlation (*R*^2^ values) are shown. **(A)** Glucose yields in saccharification with acid pretreatment. **(B)** Glucose production rates. **(C)** Xylose yields in saccharification with pretreatment. **(D)** Minor sugar yields in saccharification with pretreatment. **(E)** Glucose and xylose yields in saccharification without pretreatment. **(F)** Minor sugar yields in saccharification without pretreatment. NP, not pretreated; PT, with acid pretreatment; EH, yields in enzymatic hydrolysis; PL, yields in pretreatment liquid; and EH + PL, total yields. *R*^2^ values significant at *p* < 10% (according to ANOVA *F*-tests) are emboldened.

Experiments with *WP:AnAXE1* plants enabled direct comparison of saccharification parameters for wood lignocellulose from greenhouse-grown trees and stem lignocellulose from five-year-old field-grown trees. Linear regressions were fitted for all saccharification parameters, as shown (with *R*^2^ coefficients of determination) in Figures 7A–F. The correlations were positive for all parameters, except two: glucose yields in PL and xylose yields in EH (which were very low and not important for the total yields). The highest correlations were observed for GPR and galactose yields in PT and glucose yields in NP. These findings indicate that saccharification of material from field-grown *WP:AnAXE* transgenic trees can be predicted from observations of greenhouse-grown counterparts.

### Biomass Characteristics of Selected Lines Grown in the Field

For detailed characterization of properties of lignocellulose from the field-grown plants carrying each of the constructs, we selected the respective lines with the strongest deacetylation and analyzed the chemical and physical properties of their ground stem material. One of the *WP:GUS* lines was also analyzed since it exhibited distinct differences from WT plants in saccharification parameters. Analysis of the non-cellulosic sugars revealed increases in mol % of mannose from *35SAnAXE1*, galactose from *35S:RWA-CD* and *35S:AnAXE1*, and galacturonic acid from *WP:GUS* than from WT (Table 4; Supplementary Table S6). Total matrix sugar contents were decreased in *35S:RWA-CD*, *35S:HjAXE*, and *WP:GUS* lines. Crystalline cellulose content and porosity of the transgenics did not differ significantly from the WT plants. Some changes were recorded in soluble fructose contents, which were reduced in all lines but *35S:HjAXE*, whereas soluble sucrose and glucose, as well as starch, contents did not differ between the transgenic and WT plants.

**Table 4:** Wood traits of selected lines grown in the field.

Traits	*35S:RWA-CD* line 21	*35S:AnAXE1* line 4	*35S:HjAXE* line 13	*WP:AnAXE1* line 5	*WP:HjAXE* line 14C	*WP:GUS* line 27	WT
Ara (Mol %)	13.5 ± 1.2	11.8 ± 0.6	10.9 ± 0.9	12.2 ± 1.4	12.4 ± 0.5	11.2 ± 0.9	13.2 ± 0.2
Rha (Mol %)	3.96 ± 0.21	3.35 ± 0.18	3.45 ± 0.25	3.79 ± 0.2	3.60 ± 0.02	3.44 ± 0.2	3.85 ± 0.10
Fuc (Mol %)	0.43 ± 0.001	0.44 ± 0.01	0.37 ± 0.03	0.40 ± 0.03	0.39 ± 0.02	0.36 ± 0.01	0.41 ± 0.01
Xyl (Mol %)	46.1 ± 2.5	52.5 ± 2.1	51.2 ± 2.2	49.1 ± 2.4	50.4 ± 0.2	55.6 ± 3.0	49.2 ± 0.5
Man (Mol %)	2.05 ± 0.07	**2.42 ± 0.10** ^***^	2.17 ± 0.04	1.97 ± 0.02	2.05 ± 0.05	2.14 ± 0.12	1.94 ± 0.02
MeGlcA (Mol %)	0.31 ± 0.02	0.34 ± 0.02	0.31 ± 0.05	0.31 ± 0.02	0.31 ± 0.01	0.41 ± 0.02	0.33 ± 0.01
Gal (Mol %)	**3.49 ± 0.02** ^*^	**3.78 ± 0.07** ^**^	3.07 ± 0.28	2.93 ± 0.19	3.16 ± 0.08	2.69 ± 0.09	2.96 ± 0.1
GalA (Mol %)	18.7 ± 1.64	12.7 ± 1.6	15.3 ± 0.95	17.0 ± 1.4	16.0 ± 0.4	**11.7 ± 2.59** ^*^	17.0 ± 0.4
Glc (Mol %)	11.3 ± 0.5	12.5 ± 0.2	13.0 ± 0.7	12.0 ± 0.95	11.6 ± 0.4	12.3 ± 0.6	11.0 ± 0.3
GlcA (Mol %)	0.17 ± 0.03	0.13 ± 0.01	0.17 ± 0.04	0.20 ± 0.04	0.15 ± 0.01	0.12 ± 0.01	0.17 ± 0.01
Total TMS sugar yield (mg/g)	**234 ± 3** ^*^	240 ± 3	**234 ± 7** ^*^	246 ± 4	261 ± 5	**231 ± 8** *	254 ± 3
Cellulose (mg/g)	338 ± 8	354 ± 8	332 ± 14	353 ± 22	349 ± 15	373 ± 6	357 ± 10
BET surface area m^2^/g	1.38 ± 0.05	1.23 ± 0.13	1.42 ± 0.06	1.44 ± 0.14	1.29 ± 0.08	1.09 ± 0.12	1.09 ± 0.04
Soluble fructose (%)	**0.24 ± 0.01** ^*^	**0.13 ± 0.02** ^***^	**0.37 ± 0.10** ^**^	**0.18 ± 0.01** ^***^	**0.13 ± 0.01** ^***^	**0.22 ± 0.01** ^**^	0.29 ± 0.01
Soluble sugars and starch (%)	**0.49 ± 0.01** ^**^	**0.35 ± 0.04** ^***^	0.64 ± 0.03	**0.43 ± 0.02** ^***^	**0.40 ± 0.02** ^***^	**0.50 ± 0.03** ^**^	0.60 ± 0.01

* *p* < 5%;

** *p* < 1%;

*** *p* < 0.1%;

Means ± *SE*, *N* = 3 for transgenic lines and six for WT. Emboldened values significantly differ from WT values according to Dunnett’s test.

### Biomass Characteristics Correlating With Saccharification

**Figure 8:**
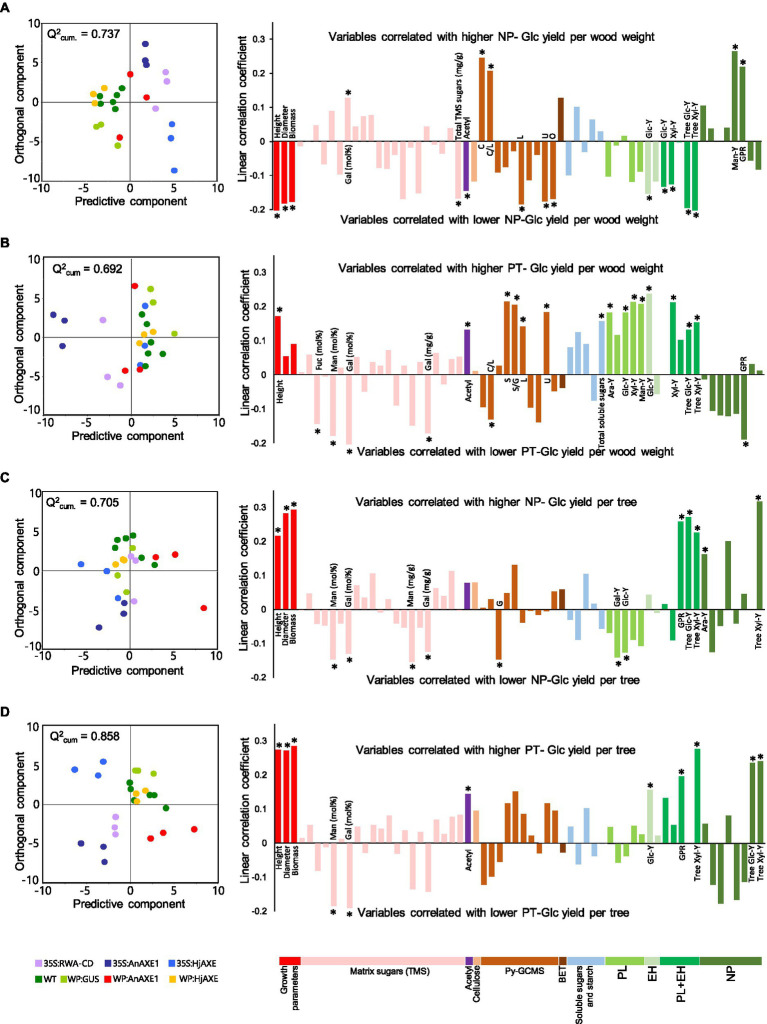
Contributions of indicated variables to predicted glucose yield in saccharification. Modeled contributions to: **(A)** glucose yield per unit wood weight in saccharification without pretreatment (NP), **(B)** glucose yield per unit wood weight in saccharification with acid pretreatment (PT), **(C)** “tree glucose yield” in NP, and **(D)** “tree glucose yield” in PT. For each model, an orthogonal projections to latent structures scatter plot showing the distribution of aspen samples (dots) colored according to constructs is presented on the left. The horizontal axes show spreads of samples according to the predictive component while the vertical axes show spreads along the orthogonal component not correlating with glucose yields. Orthogonal projections to latent structures loading plots of the respective models are shown on the right. Loadings marked with stars are important for the predictive component (VIP_predictive_ >1) and significantly contribute to the total model (VIP_total_ – 95% confidence interval > 0). PL, pretreatment liquid; EH, enzymatic hydrolysis; Y, yield; and GPR, glucose production rate.

Multivariate analysis was subsequently performed using OPLS and VIP estimates were calculated for each considered variable (Galindo-Prieto et al., 2014) to identify key biomass parameters determining saccharification yields of the field-grown trees. Orthogonal projections to latent structures models and VIP estimates are presented in Supplementary Table S7, while scatter plots and significant loadings are shown in Figure 8. Glucose yield per g of wood in NP (Figure 8A) was positively affected by matrix galactose units and carbohydrate contents, and negatively affected by plant height, stem diameter and biomass, and total sugars (detected following trimethylsilylation), acetyl, and lignin contents (Figure 8A).

In practice, it is important to identify variables that influence sugar yields of a tree, thus taking into account growth as well as biomass saccharification efficiency. To estimate “tree glucose yields,” we took into account individual trees’ stem volumes (Derba-Maceluch et al., 2020) and a fixed wood basic specific gravity, as specified in *Materials and Methods*. When glucose yields per tree in NP were modeled (Figure 8C), the growth parameters were found to be important and positively correlated, while matrix-derived galactose and mannose, and G-lignin contents were negatively correlated with these yields.

Glucose yield per g of wood in PT depended on different variables from those in NP. Height, acetyl contents, and total lignin contents were positively correlated with glucose yield in PT, whereas matrix galactose content was negatively correlated (Figure 8B). In addition, new important variables were identified, including matrix-derived fucose and mannose contents (negatively correlated with glucose yield in PT) and the S/G ratio as well as both soluble sugar and S lignin contents (positively correlated).

“Tree glucose yield” in PT (Figure 8D) was highly positively correlated with the growth parameters and acetyl content, but negatively correlated with matrix galactose and mannose contents. Without pretreatment, “tree glucose yield” (Figure 8C) was similarly positively correlated with growth parameters, but negatively correlated with matrix galactose and mannose, and G lignin, contents.

Similar OPLS-VIP modeling was applied to data on the greenhouse-grown *WP:AnAXE1* lines 5 and 10, and WT plants, to identify the most important variables affecting their saccharification yields. The analysis revealed a partially overlapping set of variables with those discussed above for the field-grown trees. The yield of glucose in NP was positively affected by soluble glucose, fructose and sucrose contents, as well as contents of matrix galactose and glucose units, but negatively correlated with contents of xylose units, 4-*O*-methyl-glucuronic acid units, acetyl groups, H lignin, crystalline cellulose, and the lignin S/G ratio (Supplementary Table S8). Orthogonal projections to latent structures-variable influence on projection analysis of “tree glucose yield” in NP revealed similar variables to those found to be important for the glucose yields per g wood prediction, with a few exceptions (Supplementary Table S8). These included, as expected, tree biomass and lignocellulose carbohydrate content (positively related) and lignin content (negatively related). The variables that ceased to be important included matrix xylose and Me-glucuronic acid units, crystalline cellulose, and acetyl contents.

Glucose yields per g wood in PT were positively correlated with contents of glucose and galactose units in the matrix, and negatively correlated with contents of xylose units and Me-glucuronic acid units in the matrix, similar to the results for such yields per g wood without pretreatment (Supplementary Table S8). For the “tree glucose yields” in PT, the stem diameter and biomass, matrix rhamnose units, and soluble sugar contents were also important and positively correlated, while the S lignin content and S/G ratio were important and negatively correlated with glucose yields.

## Discussion

### Wood Phenotypes of Acetylation-Reduced Lines Are Largely Maintained in Field Conditions

Wood properties of transgenic trees are typically evaluated using greenhouse-grown trees. However, improvements seen in greenhouse conditions are not always maintained in the field. For example, poplar lines expressing fungal xyloglucanase, which had increased cellulose content and strongly improved saccharification yields when grown in the greenhouse (Park et al., 2004; Kaida et al., 2009) lost these characteristics in field conditions (Taniguchi et al., 2012). The genetically modified lines studied here, whose main wood phenotypic characteristics are a reduction in wood acetyl content, maintained this trait for at least 5 years of growth in the field (Figure 3). Similar to results with greenhouse-grown trees in which different strategies were used to moderately decrease acetyl content (Table 3; Ratke et al., 2015; Pawar et al., 2017b; Wang et al., 2020), no major differences in carbohydrate or lignin contents were detected between these transgenic trees and WT trees when grown in the field. The only exception was that *WP:HjAXE* lines had slightly increased lignification in the field, due to increases in G-lignin units (Table 2), but not in the greenhouse (Wang et al., 2020). So with exception of this construct, which showed additional wood chemistry phenotype not observed in the greenhouse conditions, the genetically induced alterations in wood chemistry seem to be maintained in the field conditions.

### Field-Grown Acetyl-Reduced Lines Have Improved Sugar Yields in Saccharification Without Pretreatment

Most lines with reduced acetyl contents in the field provided higher glucose yields than WT in NP, but no improvement in PT (Figures 4–6). These results largely confirmed the saccharification phenotypes of greenhouse-grown trees analyzed here (Figures 2, 7) and previously (Pawar et al., 2017a,b; Wang et al., 2020). The findings support the hypothesis that sufficiently severe acid pretreatment largely eliminates the recalcitrance caused by the presence of acetyl groups. Thus, the main practical advantage of using such genetically modified lines may be the possibility of reducing pretreatment severity.

Although glucose yields in NP were negatively correlated with acetyl content, as revealed by the OPLS modeling (Supplementary Table S8; Figure 8), the relationship between glucose yield in NP and acetyl content is not linear. For example, *WP:AnAXE1* line 10 showed less improvement in glucose yields than line 1 (Figures 2, 4) despite stronger deacetylation and transgene expression (Figure 1). Non-linear effects of deacetylation have also been detected in *Arabidopsis* expressing *35S:AnAXE1*, as weakly expressing lines performed as well (or better) in saccharification with alkali pretreatment as the highly expressing lines (Pawar et al., 2016). Alkali pretreatment removes ester-bonded acetyl groups from xylan, so the positive effect of deacetylation on glucose yield in saccharification with alkali pretreatment indicates that the improved sugar yields depend on something other than the reduced presence of acetyl groups *per se*. An apparently plausible hypothesis is that deacetylation has helpful effects on cell wall architecture, as exemplified by increases in porosity (Table 1; Wang et al., 2020) and reductions in the size of xylan-lignin complexes (Pawar et al., 2017a). However, strong increases in the abundance of hydroxyl groups on xylan (caused by deacetylation) may increase the abundance of phenyl glycosidic, and benzyl ether types of bonds between xylan and lignin (Giummarella and Lawoko, 2016), thus decreasing the positive effect of deacetylation. The net effect of deacetylation likely depends on the final acetylation pattern along the xylan backbone (Grantham et al., 2017) and perhaps the deacetylation positions in the xylose rings. Thus, the relationship between acetylation and glucose yields in NP is complex, suggesting that other variables, some of which are mentioned above, affect recalcitrance.

### Constructs With the *35S* Promoter Provided Higher Saccharification Yields Than Those With the *WP* Promoter

Surprisingly, the *35S*-driven constructs clearly performed better than *WP*-driven constructs in terms of performance of the resulting material in NP per g of wood (Figure 8A). This cannot be explained by the degree of acetyl reduction, which was similar in lines with the two promoters (Figure 2). However, there were other subtle changes in wood composition between lines with the *35S* and *WP* promoters (Table 2), which could affect recalcitrance. *Inter alia*, the OPLS analysis revealed that carbohydrate contents and carbohydrate/lignin ratios, which were lower in *WP* lines than in *35S* lines, were strongly positively associated with glucose yields per g of wood in NP, while the total lignin content (which was higher in *WP* lines) was negatively related (Figure 8A). The higher lignin content in *WP* lines could be related to their higher growth rates, since mechanical loads are known to increase lignification (Ko et al., 2005), but other factors may play a role in the negative correlation between saccharification yields and growth parameters (Figure 8A).

When considering effects of genetic modifications intended to reduce recalcitrance, their influence on plant productivity (which is best tested in field conditions) should also be examined. Thus, we also considered glucose yields per tree (Escamez et al., 2017), which indicate net effects of recalcitrance and growth parameters. In such comparisons of the lines with reduced acetylation, the *WP:AnAXE1* lines were superior to WT, *35S:RWA-CD* and *WP:HjAXE* lines provided yields similar to WT, whereas other lines with reduced acetylation contents provided lower glucose yields per tree in NP (Figure 8C). The superior “tree glucose yields” of *WP:AnAXE1* lines were also observed in PT (Figure 8D). This must have been driven by growth parameters, because the wood of these lines did not yield more glucose in PT. Thus, the bigger gains in glucose yields in NP provided by constructs with the *35S* promoter than constructs with the *WP* promoter were offset by growth penalties, and lines with the *WP* promoter provided higher glucose yields per tree.

### Saccharification Yields From the Field-Grown Trees May Be Affected by Tension Wood

In greenhouse-grown trees, the main cell wall-related parameters that affect saccharification yields with and without pretreatment are those related to low xylan content. This was demonstrated in our study by findings in the multivariate analysis of *WP:AnAXE1* lines that parameters, such as Me-glucuronic acid, xylose, and acetate contents, were negatively correlated with glucose yield in NP (Supplementary Table S8), confirming results of earlier studies (Pawar et al., 2017a,b; Wang et al., 2020). For field-grown trees, however, the size of the trees seems to add another level of complexity, affecting saccharification in ways that depend on the presence or absence of pretreatment. For example, tree size was negatively and positively correlated with the yield of glucose in NP (Figure 8A) and PT (Figure 8B), respectively. Trees with thin stems are more flexible and likely to develop more tension wood, and thus have lower lignin and higher galactose units contents, than thicker trees (Mellerowicz and Gorshkova, 2012). Lignocellulose enriched in tension wood compared to normal wood is less recalcitrant due to its lower lignin content (Foston et al., 2011). Thus, a small tree size, low lignin, and high galactose units contents correlate with high glucose yield in NP. However, the abundance of tension wood does not influence the glucose yield in PT since the acid pretreatment is effective for lignin removal.

Interestingly, certain minor sugars seem to provide a signature of lignocellulose characteristics that correlates with large tree size and high glucose yield in PT. This includes low contents of fucose, mannose and galactose, as observed in both this study (Figure 8B) and a previous study involving a large diverse collection of *Populus* genotypes grown in the greenhouse (Escamez et al., 2017). Low mannose and galactose content is also a good predictor of “tree glucose yields” in both types of saccharification (Figures 8C,D). These two sugars reflect contents of two polymers, mannan and galactan, which are found in different cell wall locations: mannan mostly in the secondary wall and galactan in the primary wall and gelatinous layer (Mellerowicz and Gorshkova, 2012). In this study, we observed that enzymatic hydrolysis of mannan in NP was highly promoted by use of several acetyl-deficient lines (Figure 6B) and was correlated with glucose yields in NP (Figure 4B), suggesting that removal of acetyl groups from xylan increases accessibility of mannan and cellulose to hydrolytic enzymes. Saccharification yields of galactose were also elevated by use of some lines and/or conditions in the pretreatment liquid, including *WP:AnAXE1* lines grown in the greenhouse (Figure 2D) and *HjAXE*- and *GUS*-expressing lines grown in the field (Figure 6A). This indicates that galactan is a highly labile cell wall component, and thus probably associated with the presence of G-layer. Contents of these sugars are probably associated with the glucose yield in PT and “tree glucose yields” through their connection to various growth-related processes.

### *HjAXE* and *AnAXE1* Differently Affect Sugar Yields in Saccharification With and Without Acid Pretreatment

We compared effects of expressing *HjAXE* and *AnAXE1* xylan acetyl esterases of families CE5 and CE1, respectively, in field-grown trees, partly because a previous study found that trees expressing *HjAXE* ubiquitously were less productive than those expressing *AnAXE1* ubiquitously (Derba-Maceluch et al., 2020). The analysis presented here revealed fundamental differences in the changes in wood characteristics induced by the two transgenes, which have not been observed in previous studies. While both transgenes induced a similar level of deacetylation (Figure 3), *HjAXE*-expressing lines had higher lignification and S/G lignin ratios (Table 2). This was associated with differential effects of the transgenes on sugar yields in saccharification with and without pretreatment: *HjAXE* lines performed better in PT while *AnAXE1* lines performed better in NP (Figures 4–6). The most striking example was in the transgenes’ effects on xylose yield in NP, which was strongly enhanced by *AnAXE1* but reduced by *HjAXE* (Figure 5B). Since there was no major difference in xylan content between lines hosting the two constructs, as evidenced by their total sugar (detected following trimethylsilylation) content (Table 4), their differential effects in NP are presumably due to differences in xylan digestibility, probably linked to lignin levels. The mechanistic reasons for the differences in lignification induced by the two transgenes are currently unknown and could be related to various factors. *Inter alia*, the enzymes they encode differ in terms of deacetylation activity at specific positions of xylopyranose rings. *Hj*AXE has been shown to catalyze deacetylation at positions 2 and 3, including position 3 in glucuronated xylose unit (Wang et al., 2020), whereas *An* AXE1 only reportedly deacetylates at position 2 (Pawar et al., 2017a). The two enzymes could also deacetylate different xylan domains. As a member of family CE5, *Hj*AXE can deacetylate polymeric xylan, including the xylan fraction associated with cellulose microfibrils since it has a cellulose-binding domain, unlike the CE1 member, *An*AXE1 (Margolles-Clark et al., 1996). Finally, *HjAXE* could induce defense reactions in aspen, as indicated by higher frequencies of necrotic spots on leaves of *35S:HjAXE* plants (Derba-Maceluch et al., 2020).

## Perspectives

During the past three decades, several types of genetically modified plants, including xylanase- or xyloglucanase-overexpressing poplars (reviewed by Donev et al., 2018), and poplars with various kinds of lignin and cellulose or general cell wall modifications (reviewed by Chanoca et al., 2019; Bryant et al., 2020) have shown substantial improvements in wood-processing properties. However, there have been relatively few field trial studies of these lines. Moreover, in most cases reported to date, the improved processing properties of these lines were offset by poor growth in the field and their glucose yields per tree were not determined. A notable exception is the case of overexpression of the constitutively active small G protein RabG3b in poplar that affects plant growth and wood formation *via* poorly understood pathways, with positive effects on saccharification yields, according to Kim et al. (2018). In this light, the field performance of aspen plants with reduced acetylation and improved saccharification reported here is encouraging. Among several types of genetically modified plants tested in this study, those carrying at least one construct, *WP:AnAXE1*, were superior in terms of yields of glucose per tree in both kinds of saccharification. The improvements were modest, and a negative correlation was observed between glucose yields in NP and growth, suggesting an interaction with incompletely understood physiological processes. However, the finding that the lines grew well in the field conditions while retaining their improved wood-processing properties is highly encouraging and provides strong incentives for further efforts to genetically improve trees for biorefinery applications by altering acetylation.

## Data Availability Statement

The original contributions presented in the study are included in the article/Supplementary Material, and further inquiries can be directed to the corresponding author.

## Author Contributions

SP prepared wood for all analyses and performed the pyrolysis and TMS analyses. MG performed all pretreatment and saccharification, BET, soluble sugar and starch analyses. MD-M analyzed transgenic plants in the greenhouse and was responsible for cellulose and multivariate data analyses. MD-M and EJM designed the field trials and coordinated the field work. LJJ and SW supervised pretreatment and saccharification and coordinated wood analyses with EJM. EJM designed the study and wrote the manuscript with contributions from all authors. All authors contributed to the article and approved the submitted version.

## Conflict of Interest

The authors declare that the research was conducted in the absence of any commercial or financial relationships that could be construed as a potential conflict of interest.

## Publisher’s Note

All claims expressed in this article are solely those of the authors and do not necessarily represent those of their affiliated organizations, or those of the publisher, the editors and the reviewers. Any product that may be evaluated in this article, or claim that may be made by its manufacturer, is not guaranteed or endorsed by the publisher.

## Abbreviations

EH: enzymatic hydrolysate
GPR: glucose production rate
NP: saccharification without pretreatment
PL: pretreatment liquid
PT: saccharification with acid pretreatment
WT: wild type

## References

[ref1] Bar-On Y. M. Phillips R. Milo R. (2018). The biomass distribution on earth. PNAS 115, 6506–6511. 10.1073/pnas.1711842115, PMID: 29784790 PMC6016768

[ref2] Bradford M. M. (1976). A rapid and sensitive method for the quantitation of microgram quantities of protein utilizing the principle of protein-dye binding. Anal. Biochem. 72, 248–254. 10.1006/abio.1976.9999, PMID: 942051

[ref3] Bryant N. B. Pu Y. Tschaplinski T. J. Tuskan G. A. Muchero W. Kalluri U. C.. (2020). Transgenic poplar designed for biofuels. Trends Plant Sci. 25, 881–896. 10.1016/j.tplants.2020.03.008, PMID: 32482346

[ref4] Busse-Wicher M. Gomes T. C. F. Tryfona T. Nikolovski N. Stott K. Grantham N. J.. (2014). The pattern of xylan acetylation suggests xylan may interact with cellulose microfibrils as a twofold helical screw in the secondary plant cell wall of *Arabidopsis thaliana*. Plant J. 79, 492–506. 10.1111/tpj.12575, PMID: 24889696 PMC4140553

[ref5] Chanoca A. de Vries L. Boerjan W. (2019). Lignin engineering in forest trees. Front. Plant Sci. 10:912. 10.3389/fpls.2019.00912, PMID: 31404271 PMC6671871

[ref6] Chong S. L. Koutaniemi S. Virkki L. Pynnönen H. Tuomainen P. Tenkanen M. (2013). Quantitation of 4-O-methylglucuronic acid from plant cell walls. Carbohydr. Polym. 91, 626–630. 10.1016/j.carbpol.2012.08.078, PMID: 23121956

[ref7] Chong S. L. Virkki L. Maaheimo H. Juvonen M. Derba-Maceluch M. Koutaniemi S.. (2014). O-acetylation of glucuronoxylan in *Arabidopsis thaliana* wild type and its change in xylan biosynthesis mutants. Glycobiology 24, 494–506. 10.1093/glycob/cwu017, PMID: 24637390

[ref8] Chung H. J. Park S. M. Kim H. R. Yang M. S. Kim D. H. (2002). Cloning the gene encoding acetyl xylan esterase from *Aspergillus ficuum* and its expression in *Pichia pastoris*. Enzym. Microb. Technol. 31, 384–391. 10.1016/S0141-0229(02)00122-9

[ref9] Derba-Maceluch M. Amini F. Donev E. N. Pawar P. M.-A. Michaud L. Johansson U.. (2020). Cell wall acetylation in hybrid aspen affects field performance, foliar phenolic composition and resistance to biological stress factors in a construct-dependent fashion. Front. Plant Sci. 11:651. 10.3389/fpls.2020.00651, PMID: 32528503 PMC7265884

[ref10] Donev E. Gandla M. L. Jönsson L. J. Mellerowicz E. J. (2018). Engineering non-cellulosic polysaccharides of wood for the biorefinery. Front. Plant Sci. 9:1537. 10.3389/fpls.2018.01537, PMID: 30405672 PMC6206411

[ref11] Escamez S. Gandla M. L. Derba-Maceluch M. Lundqvist S. O. Mellerowicz E. J. Jönsson L. J.. (2017). A collection of genetically engineered *Populus* trees reveals wood biomass traits that predict glucose yield from enzymatic hydrolysis. Sci. Rep. 7:15798. 10.1038/s41598-017-16013-0, PMID: 29150693 PMC5693926

[ref12] Foston M. Hubbell C. A. Samuel R. Jung S. Fan H. Ding S.-Y.. (2011). Chemical, ultrastructural and supramolecular analysis of tension wood in *Populus tremula × alba* as a model substrate for reduced recalcitrance. Energy Environ. Sci. 4, 4962–4971. 10.1039/c1ee02073k

[ref13] Galbe M. Wallberg O. (2019). Pretreatment for biorefineries: a review of common methods for efficient utilisation of lignocellulosic materials. Biotechnol. Biofuels 12:294. 10.1186/s13068-019-1634-1, PMID: 31890022 PMC6927169

[ref14] Galindo-Prieto B. Eriksson L. Trygg J. (2014). Variable influence on projection (VIP) for orthogonal projections to latent structures (OPLS). J. Chemom. 28, 623–632. 10.1002/cem.2627

[ref15] Gandla M. L. Derba-Maceluch M. Liu X. Gerber L. Master E. R. Mellerowicz E. J.. (2015). Expression of a fungal glucuronoyl esterase in *Populus*: effects on wood properties and saccharification efficiency. Phytochemistry 112, 210–220. 10.1016/j.phytochem.2014.06.002, PMID: 24997793

[ref16] Gandla M. L. Mähler N. Escamez S. Skotare T. Obudulu O. Möller L.. (2021). Overexpression of vesicle-associated membrane protein *PttVAP27-17* as a tool to improve biomass production and the overall saccharification yields in *Populus* trees. Biotechnol. Biofuels 14:43. 10.1186/s13068-021-01895-0, PMID: 33593413 PMC7885582

[ref17] Gerber L. Eliasson M. Trygg J. Moritz T. Sundberg B. (2012). Multivariate curve resolution provides a high-throughput data processing pipeline for pyrolysis-gas chromatography/mass spectrometry. J. Anal. Appl. Pyrolysis 95, 95–110. 10.1016/j.jaap.2012.01.011

[ref18] Giummarella N. Lawoko M. (2016). Structural basis for the formation and regulation of lignin−xylan bonds in birch. ACS Sustain. Chem. Eng. 4, 5319–5326. 10.1021/acssuschemeng.6b00911

[ref19] Gorshkova T. Mokshina N. Chernova T. Ibragimova N. Salnikov V. Mikshina P.. (2015). Aspen tension wood fibers contain β-(1→4)-galactans and acidic arabinogalactans retained by cellulose microfibrils in gelatinous walls. Plant Physiol. 169, 2048–2063. 10.1104/pp.15.00690, PMID: 26378099 PMC4634055

[ref20] Grantham N. J. Wurman-Rodrich J. Terrett O. M. Lyczakowski J. J. Stott K. Iuga D.. (2017). An even pattern of xylan substitution is critical for interaction with cellulose in plant cell walls. Nat. Plants 3, 859–865. 10.1038/s41477-017-0030-8, PMID: 28993612

[ref21] Hendriks J. H. Kolbe A. Gibon Y. Stitt M. Geigengerger P. (2003). ADP-glucose pyrophosphorylase is activated by posttranslational redox modification in response to light and to sugars in leaves of *Arabidopsis* and other plant species. Plant Physiol. 133, 838–849. 10.1104/pp.103.024513, PMID: 12972664 PMC219057

[ref22] Jönsson L. J. Alriksson B. Nilvebrant N.-O. (2013). Bioconversion of lignocellulose: inhibitors and detoxification. Biotechnol. Biofuels 6:16. 10.1186/1754-6834-6-16, PMID: 23356676 PMC3574029

[ref23] Kaida R. Kaku T. Baba K. Oyadomari M. Watanabe T. Nishida K.. (2009). Loosening xyloglucan accelerates the enzymatic degradation of cellulose in wood. Mol. Plant 2, 904–909. 10.1093/mp/ssp060, PMID: 19825667

[ref24] Kim N. Y. Jeon H. S. Lee M.-H. Cha A. Lee D. S. Lee H. J.. (2018). Field evaluation of transgenic poplars expressing the constitutively active small G protein for improved biomass traits. Biomass Bioenergy 109, 16–22. 10.1016/j.biombioe.2017.12.015

[ref25] Ko J.-H. Han K.-H. Park S. Yang J. (2004). Plant body weight-induced secondary growth in arabidopsis and its transcription phenotype revealed by whole-transcriptome profiling. Plant Physiol. 135, 1069–1083. 10.1104/pp.104.038844, PMID: 15194820 PMC514141

[ref26] Lee C. H. Teng Q. Zhong R. Q. Ye Z. -H. (2011). The four *Arabidopsis* reduced wall acetylation genes are expressed in secondary wall-containing cells and required for the acetylation of xylan. Plant Cell Physiol. 52, 1289–1301. 10.1093/pcp/pcr075, PMID: 21673009

[ref27] Manabe Y. Verhertbruggen Y. Gille S. Harholt J. Chong S.-L. Pawar P. M. A.. (2013). Reduced wall acetylation proteins play vital and distinct roles in cell wall O-acetylation in Arabidopsis. Plant Physiol. 163, 1107–1117. 10.1104/pp.113.225193, PMID: 24019426 PMC3813637

[ref28] Margolles-Clark E. Tenkanen M. Söderlund H. Penttilä M. (1996). Acetyl xylan esterase from *Trichoderma reesei* contains an active-site serine residue and a cellulose-binding domain. Eur. J. Biochem. 237, 553–560. 10.1111/j.1432-1033.1996.0553p.x, PMID: 8647098

[ref29] Martinez-Abad A. Giummarella N. Lawoko M. Vilaplana F. (2018). Differences in extractability under subcritical water reveal interconnected hemicellulose and lignin recalcitrance in birch hardwoods. Green Chem. 20, 2534–2546. 10.1039/C8GC00385H

[ref30] McCann M. C. Carpita N. C. (2015). Biomass recalcitrance: a multi-scale, multi-factor and conversion-specific property. J. Exp. Bot. 66, 4109–4118. 10.1093/jxb/erv267, PMID: 26060266

[ref31] Mellerowicz E. J. Gorshkova T. (2012). Tensional stress generation in gelatinous fibres: a review and possible mechanism based on cell wall structure and composition. J. Exp. Bot. 63, 551–565. 10.1093/jxb/err339, PMID: 22090441

[ref32] Park Y. W. Baba K. Furuta Y. Iida I. Sameshima K. Arai M.. (2004). Enhancement of growth and cellulose accumulation by overexpression of xyloglucanase in poplar. FEBS Lett. 564, 183–187. 10.1016/S0014-5793(04)00346-1, PMID: 15094064

[ref33] Pauly M. Ramírez V. (2018). New insights into wall polysaccharide O-acetylation. Front. Plant Sci. 9:1210. 10.3389/fpls.2018.01210, PMID: 30186297 PMC6110886

[ref34] Pawar P. M.-A. Derba-Maceluch M. Chong S.-L. Gandla M. L. Bashar S. S. Sparrman T.. (2017a). *In muro* deacetylation of xylan increases lignin extractability and improves saccharification of aspen wood. Biotechnol. Biofuels 10:98. 10.1186/s13068-017-0782-4, PMID: 28428822 PMC5397736

[ref35] Pawar P. M.-A. Derba-Maceluch M. Chong S.-L. Gómez L. D. Miedes E. Banasiak A.. (2016). Expression of fungal acetyl xylan esterase in *Arabidopsis thaliana* improves saccharification of stem lignocellulose. Plant Biotechnol. J. 14, 387–397. 10.1111/pbi.12393, PMID: 25960248 PMC11389080

[ref36] Pawar P. M.-A. Ratke C. Balasubramanian V. B. Chong S.-L. Gandla M. L. Adriasola M.. (2017b). Downregulation of RWA genes in hybrid aspen affects xylan acetylation and wood processing properties. New Phytol. 214, 1491–1505. 10.1111/nph.14489, PMID: 28257170

[ref38] Pfaffl M. W. (2001). A new mathematical model for relative quantification in real-time RT-PCR. Nucleic Acids Res. 29:e45. 10.1093/nar/29.9.e45, PMID: 11328886 PMC55695

[ref39] Ramirez V. Xiong G. Mashiguchi K. Yamaguchi S. Pauly M. (2018). Growth- and stress-related defects associated with wall hypoacetylation are strigolactone-dependent. Plant Direct 2:e00062. 10.1002/pld3.62, PMID: 31245725 PMC6508513

[ref40] Ratke C. Pawar P. M.-A. Balasubramanian V. K. Naumann M. Lönnäs Duncranz M. Derba-Maceluch M.. (2015). *Populus GT43* family members group into distinct sets required for primary and secondary wall xylan biosynthesis and include useful promoters for wood modification. Plant Biotechnol. J. 13, 26–37. 10.1111/pbi.12232, PMID: 25100045

[ref41] Roach M. Gerber L. Sanquist D. Gorzsas A. Hedenstrom M. Kumar M.. (2012). Fructokinase is required for carbon portioning to cellulose in aspen wood. Plant J. 70, 967–977. 10.1111/j.1365-313X.2012.04929.x, PMID: 22288715

[ref42] Smith A. M. Zeeman S. C. (2006). Quantification of starch in plant tissues. Nat. Protoc. 1, 1342–1345. 10.1038/nprot.2006.232, PMID: 17406420

[ref43] Stitt M. Lilley R. M. Gerhardt R. Heldt H. W. (1989). Metabolite levels in specific cells and subcellular compartments of plant leaves. Methods Enzymol. 174, 518–552. 10.1016/0076-6879(89)74035-0

[ref44] Taniguchi T. Konagaya K. Kurita M. Takata N. Ishii K. Kondo T.. (2012). Growth and root sucker ability of field-grown transgenic poplars overexpressing xyloglucanase. J. Wood Sci. 58, 550–556. 10.1007/s10086-012-1281-7

[ref37] Urbanowicz B. R. Peňa M. J. Moniz H. A. Moremen K. W. York W. S. (2014). Two Arabidopsis proteins synthesize acetylated xylan *in vitro*. Plant J. 80, 197–206. 10.1111/tpj.12643, PMID: 25141999 PMC4184958

[ref45] Wang Z. Pawar P. M.-A. Derba-Maceluch M. Hedenström M. Chong S.-L. Tenkanen M.. (2020). Hybrid aspen expressing a carbohydrate esterase family 5 acetyl xylan esterase under control of a wood-specific promoter shows improved saccharification. Front. Plant Sci. 11:380. 10.3389/fpls.2020.00380, PMID: 32322259 PMC7156598

[ref46] Wang Z. Winestrand S. Gillgren T. Jönsson L. J. (2018). Chemical and structural factors influencing enzymatic saccharification of wood from aspen, birch and spruce. Biomass Bioenergy 109, 125–134. 10.1016/j.biombioe.2017.12.020

[ref47] Xie F. Xiao P. Chen D. Xu L. Zhangmi B. (2012). mIRDeepFinder: a miRNA analysis tool for deep sequencing of plant small RNAs. Plant Mol Biol. 80, 75–84. 10.1007/s11103-012-9885-2, PMID: 22290409

[ref48] Xiong G. Cheng K. Pauly M. (2013). Xylan O-acetylation impacts xylem development and enzymatic recalcitrance as indicated by the Arabidopsis mutant tbl29. Mol Plant 6, 1373–1375. 10.1093/mp/sst014, PMID: 23340742

[ref49] Yoo C. G. Yang Y. Pu Y. Meng X. Muchero W. Yee K. L.. (2017). Insights of biomass recalcitrance in natural *Populus trichocarpa* variants for biomass conversion. Green Chem. 19, 5467–5478. 10.1039/C7GC02219K

[ref50] Zhong R. Cui D. Richardson E. A. Phillips D. R. Azadi P. Lu G.. (2020). Cytosolic acetyl-CoA generated by ATP-citrate Lyase is essential for acetylation of cell wall polysaccharides. Plant Cell Physiol. 61, 64–75. 10.1093/pcp/pcz178, PMID: 31503286

[ref51] Zhong R. Cui D. Ye Z.-H. (2017). Regiospecific acetylation of xylan is mediated by a group of DUF231-containing O-acetyltransferases. Plant Cell Physiol. 58, 2126–2138. 10.1093/pcp/pcx147, PMID: 29059346

